# Community Health Volunteers’ experiences of implementing COVID-19 vaccine education and promotion in Kenya: a qualitative descriptive study

**DOI:** 10.3389/fpubh.2024.1406959

**Published:** 2024-07-10

**Authors:** Constance S. Shumba, Peterson Kiraithe, Isabel Kambo, Sheila Shaibu

**Affiliations:** ^1^Division of Epidemiology and Social Sciences, Institute for Health and Equity, Medical College of Wisconsin, Milwaukee, WI, United States; ^2^School of Nursing and Midwifery, Aga Khan University, Nairobi, Kenya

**Keywords:** Community Health Volunteers, COVID-19 vaccination, vaccine uptake, pandemic, Kenya

## Abstract

**Background:**

Vaccination was a key measure in the COVID-19 pandemic response, though much work was needed to promote vaccine uptake and acceptance. In Kenya, Community Health Volunteers (CHVs) played a key role in vaccine education and promotion. We conducted this study to explore CHVs’ experiences of implementing COVID-19 vaccine education and promotion during the pandemic to increase COVID-19 vaccine uptake in two areas of Kenya.

**Methods:**

In a qualitative descriptive study, we conducted 30 structured in-depth interviews with 20 CHVs and 10 Community Health Assistants from rural Kilifi County and Kangemi, an urban informal settlement of Nairobi County in Kenya between April 2022 and July 2022.

**Findings:**

Thematic analysis generated five key themes in relation to CHVs’ experiences of implementing COVID-19 vaccine education and promotion: Five key themes emerged regarding CHVs’ experiences of implementing COVID-19 vaccine education and promotion: (1) vaccine preferences influenced acceptance, (2) the fear of side effects was a barrier, (3) misinformation was widespread (4) lack of trust in government and politicization of vaccines was a barrier, and (5) CHVs’ efforts were a facilitator to increased uptake.

**Conclusion:**

Extensive community outreach from CHVs contributed to the high uptake of primary vaccines and boosters during the COVID-19 pandemic. CHVs acting as role models by receiving vaccinations first was particularly important in influencing communities to accept vaccinations. Findings provide evidence for prioritizing CHVs in the planning and implementation of future vaccination initiatives in Kenya and other countries.

## Introduction

COVID-19 is a disease caused by the SARS-CoV-2 coronavirus and since December 2019 when the first case was recorded in Wuhan, China, there have been over 760 million cases and 6.9 million deaths globally (1, 2). Since the beginning of the pandemic, Kenya registered, 344,094 confirmed cases and 5,689 deaths (3). In 2020 and 2021, there was a global focus on the development and deployment of vaccines to reduce COVID-19-related illness and mortality (4). There was a rapid development of COVID-19 vaccines to provide protection against severe illness and death, with more than 13 billion vaccine doses administered worldwide by June 2023 (1). The roll-out of the main types of COVID-19 vaccines in Kenya, namely Pfizer, Johnson and Johnson, Moderna, AstraZeneca and Sinopharm, commenced in March 2021, with the goal of attaining 100% vaccination coverage by December 2022 (5). However, by March 2023, a total of 23,359,310 COVID-19 vaccine doses had been administered in the country, with 14,317,039 people receiving at least one dose, and only 26.63% of the population having received at least one dose thereby falling short of the target (6). The poor vaccine coverage was largely attributed to vaccine inequity issues as countries in Africa did not have timely access to COVID-19 vaccines, had limited capacity for regional vaccine manufacturing thereby relying on foreign production, coupled with challenges in distribution and utilization of the vaccines once they were accessible (7, 8).

Vaccination was a vital measure for reducing virus transmission and minimizing public health impact, however, to be effective, the immunization program required high uptake and acceptance rates. While the success of vaccine deployment hinged on a wide range of factors, last-mile delivery and roll-out were particularly important. Community Health Workers (CHWs) were critical in introducing vaccines to local communities, given their understanding of these communities and their extensive knowledge of other vaccination drives (4). From the outset of the vaccination roll-out, it was evident that a lot of work would be required from CHWs to boost trust, acceptance, and uptake. CHWs have been defined previously as lay health workers who have some basic training to promote health in their communities and often are not salaried (9). Strengthening community health systems that include several cadres of community health workers, has become a pertinent area of focus in supporting achievement of universal health coverage in sub-Saharan Africa (9). There are ongoing efforts to formally standardize their training and professionalize and remunerate them (10). The roles of CHWs differ across contexts but typically will involve preventive and promotive services, community drug distribution, diagnosis of some illnesses, referrals, and health and vital statistics reporting (9). CHWs are trusted by their communities and often the first line of contact with the health system, acting as a link between their communities and health facilities. In Kenya, CHWs who are referred to as Community Health Volunteers (CHVs) receive basic training but are not considered healthcare professionals although they are engrained into Kenya’s health system with a guiding national framework (11, 12). The CHVs are drawn from the local communities, play a crucial role in increasing access to health services among marginalized population, and are supervised by Community Health Assistants (12, 13). Due to their established roles mobilizing communities to promote child health and immunization, there was an expectation/a default position that CHVs would also play a major role in promoting uptake of COVID-19 vaccines in Kenya (12).

In Kenya, Community Health Volunteers (CHVs) played a key role in COVID-19 vaccine outreach and as part of the frontline health workforce, they promoted uptake through community engagement and mobilization, but their perspectives are underrepresented in the literature on their experiences and contributions to the pandemic response. There is evidence on how the knowledge, attitudes, and perceptions of CHWs regarding COVID-19 vaccination influenced vaccine hesitancy (12, 14, 15). However, less is known about the experiences of CHWs in conducting COVID-19 vaccination outreach in their communities. The capacity to implement effective risk communication and community engagement strategies including vaccine education and promotion in a pandemic context hinge on a strong comprehension of the critical strategies deployed by CHVs in their communities, barriers, and enablers, but this is understudied. Understanding these experiences is crucial for improving vaccination uptake and addressing vaccine hesitancy among communities. We conducted this study to explore CHVs’ experiences of implementing COVID-19 vaccine education and promotion during the pandemic to increase COVID-19 vaccine uptake in two areas of Kenya.

## Methods

### Study design and setting

This was a qualitative descriptive study conducted between April 2022 and July 2022 in two areas of Kenya. In this paper, we focus on the specific experiences of CHVs providing COVID-19 vaccine education and promotion to increase vaccination uptake. This study was undertaken as part of a broader study aimed investigating the roles of CHVs during the COVID-19 pandemic in rural and urban Kenya. The qualitative descriptive design was chosen because it enabled examination of how the CHVs experienced promoting COVID-19 vaccine uptake in their communities in Kenya, an important topic sparsely documented in the literature on COVID-19 responses thereby contributing to the body of knowledge (16). Further, this design was preferred to provide the foundational understanding and set the basis for future studies among CHVs and their communities on COVID-19 vaccination. The qualitative descriptive design is a distinctive component of qualitative research that is in itself valuable especially when direct descriptions of phenomena including who, what and where are desired by the researcher (17).

We chose two distinct geographical settings to capture the diversity of experiences in an urban and rural context and compare. The first, Kilifi County, in particular Kaloleni and Rabai Sub-Counties are rural areas with an estimated population of 352,175 of whom 70% live below the poverty line (18). They are some of the poorest areas in the country and heavily reliant on subsistence agriculture and tourism, with limited health infrastructure and long distances to primary health care facilities (13). The second area, Kangemi, is a low-income informal settlement in the western part of Nairobi County, the capital of Kenya, with an estimated population of 100,000 (19, 20).

### Sampling and recruitment

We purposively recruited a total of 30 participants comprising 20 CHVs and 10 Community Health Assistants (CHAs), with 10 and five (5) from each county, respectively. A total of 30 participants was sufficient to achieve saturation, whereby no new themes emerged on the phenomenon of the CHVs experiences in promoting COVID-19 vaccine uptake (21). Community Health Assistants supervise CHVs within community health units, which each comprise an average of 1,000 households and 5,000 people within a geographically defined area. Each unit is aligned to an administrative sub-location with 10 CHVs and specified health facilities (22). The CHAs were included as participants in the study in addition to CHVs, given their supervisory role of 10 CHVs, their understanding of the roles of multiple CHVs and how these evolved during the pandemic period. Their perspectives were critical to aiding understanding of how the experiences were shaped among several CHVs, thereby complementing the study’s objective.

### Data collection and interview process

We held entry meetings with county leadership teams and approached CHAs in participating counties who then introduced us to the CHVs in their respective areas. We informed the CHAs and CHVs about the study, emphasized confidentiality and voluntary participation, as well as risks and benefits of study. We asked those who were willing to participate to contact the study team to arrange a phone interview. We developed structured in-depth interview guides in English and translated them to Kiswahili. The interviews were all telephonically conducted by PK, a trained and experienced Research Assistant and the recordings and transcripts were checked by SS and CS, ensuring consistency in data collection. We pilot-tested the interview guides with two participants to identify and remedy any problems. The interviews lasted about an hour and were concluded with a summary and debrief of the issues discussed. Key questions were: What were the communities’ attitudes toward the COVID-19 vaccines? What are some of the reasons why community members accepted to be vaccinated? What are some of the reasons community members gave for refusing to be vaccinated? How was the vaccine uptake in your area? What do you think were the factors that influenced the vaccination coverage rate that you attained in your community? Were there any specific challenges you faced as a CHV while promoting COVID-19 vaccine uptake?

### Data management and analysis

All interviews were audio-recorded and transcribed from Kiswahili into English and back translated. Transcripts were checked against the audio recordings for quality checks and de-identified prior to uploading to a secure database at the Aga Khan University, School of Nursing and Midwifery. We imported and analyzed the data with NVIVO-12 software and utilized a thematic inductive approach (23), which involved development of a coding framework with themes and categories. We employed data source triangulation, by examining the data from both CHVs and CHAs and checking for concurrence or divergence of the information, enhancing our understanding of CHVs’ experiences in promoting COVID-19 vaccine uptake (24). Transcripts were coded independently by PK and CS and reviewed by SS. Where there was lack of agreement on the coding, CS and SS re-examined the transcripts and reached consensus through discussion. The study team also held a final in-depth discussion and review of key themes to ensure agreement on the codebook.

### Ethics

We obtained ethical approval from both the Aga Khan University Kenya’s Institutional Scientific and Ethics Review Committee [Ref: 2020/IERC-89(v3)] and the National Commission for Science Technology and Innovation (EOP/NMS/HS/088). We also sought and obtained permission to conduct the study from county governments, local leaders, and gatekeepers. Further, we obtained voluntary informed consent from all CHAs and CHVs who participated in the study. Participant privacy and confidentiality were assured during data collection as no specific identifiers were recorded and all transcripts were de-identified granting anonymity, assigned codes and could not be linked bank to the participants (25). None of the analysis and study reports included any identifying information on individual participants. Further, the data was stored in a secure password protected database accessed only by the research team.

## Results

### Characteristics of CHVs and CHAs

Our study had a wide range of participants ages 20–70 years, with 65% between 20–40 years. Half (50%) of the participants had completed primary and secondary level education while the rest had post-secondary education. Most (65%) had 6–20 years’ experience as CHVs while the rest had 1–5 years’ experience. Household coverage for CHVs ranged from 25–100 households, with most (60%) covering 76–100 households as part of their duties. Our analysis indicated that CHAs and CHVs from the two regions played a crucial role in promoting vaccine uptake and acceptance during the COVID-19 pandemic. Five key themes emerged regarding CHVs’ experiences of implementing COVID-19 vaccine education and promotion: (1) vaccine preferences influenced acceptance, (2) the fear of side effects was a barrier, (3) misinformation was widespread (4) lack of trust in government and politicization of vaccines was a barrier, and (5) CHVs’ efforts were a facilitator to increased uptake. Data are presented under each theme and sub-theme (Table 1) with supporting de-identified quotes from CHVs and CHAs.

**Table 1:** Identified themes and sub-themes.

Themes	Sub-themes
1. Vaccine preferences influenced acceptance	1.1 Preference for the Moderna vaccine 1.2 Preference linked to side effects 1.3 Uncertainty of suitability of vaccines for individuals
2. Fear of side effects was a barrier	2.1 Lost time from work/income loss 2.2 Reported side effects 2.3 Delayed acceptance to ascertain vaccine impact on others
3. Misinformation was widespread	3.1 Vaccine a cause of infertility 3.2 Interference with well-being and death 3.3 Non-existence of COVID-19
4. Lack of trust in government hindered uptake	4.1 Rumors of voting through the COVID-19 vaccine 4.2 Government accused of selling citizens
5. CHVs’ efforts were a facilitator to increased uptake	5.1 CHVs prioritized for vaccination 5.2 CHVs increased vaccination uptake

### Theme 1: vaccine preferences influenced acceptance

In the narratives of the CHVs, as COVID vaccines became available in the country and they provided vaccine education and promotion, it was apparent that preferences had already been formed. Participants described how Moderna (mRNA vaccine) was preferred over AstraZeneca, mostly attributed to concerns surrounding safety, effectiveness, side effects and individual suitability of vaccines. This theme came up consistently in the informal settlements of Kangemi, in comparison to rural Kilifi where this did not emerge as a concern.

#### Sub-theme 1.1: preference for the Moderna vaccine

As COVID-19 vaccines became available, many expressed uneasiness and confusion. CHVs reported that there was a strong preference for the Moderna vaccine over AstraZeneca. Some people thought the AstraZeneca vaccine would be harmful, influenced by false information and rumors regarding safety. For instance, one rumor circulated that the AstraZeneca vaccine was causing people to die. Despite such rumors, CHVs worked hard to convince their communities to take up the AstraZeneca vaccine due to Moderna stockouts.

Respondents indicated a strong preference for the Moderna vaccine which they believed was safe and effective compared to other accessible alternatives. However, some people made decisions based on vaccine accessibility, consenting to receive the AstraZeneca vaccine for their second dose when Moderna was unavailable.

*At first, people were saying if you were vaccinated with AstraZeneca you will die… most, people had fear in those types of names… One could say if you are injected this one you die, this one you get drowsy… They had different opinions, according to the types of vaccines they were using.* **Kangemi** CHV **01 Script**

*… I know about the households I am in charge of. … It reached a point, and it was said that Moderna has finished and not available… I went to inquire from the facility, and I was told that for ones that took Moderna for the first dose, since Moderna was out of stock, let them take AstraZeneca for the second dose, and that’s what I did.* **Kangemi** CHV **09 Script**

#### Sub-theme 1.2: preference linked to side effects

Vaccine preferences were also linked to perceived side effects of specific vaccines and the severity of these effects. For instance, one CHV reported that the Johnson and Johnson vaccine was believed to cause insomnia. Communities held several incorrect beliefs about vaccines’ adverse effects which were often based on rumors. Conversely, there was a preference for vaccines with perceived lesser or more manageable side effects.

*It is just a myths and misconception thing, the myths that they were discussing. You find people where they meet saying, you know, I was vaccinated with Johnson and Johnson, it is bad and you cannot fall asleep. You find another one saying I was vaccinated with the Moderna. Moderna makes one go to sleep.* **Kangemi** CHV **04 Script**

#### Sub-theme 1.3: uncertainty of suitability of vaccines for individuals

It was reported that many community members wanted assurance on the suitability of specific vaccines for them as individuals. Findings suggested that people sought individualized information and counseling to make knowledgeable decisions about vaccination. However, it was generally not possible to provide such advice. Variables such as age, allergies, pre-existing health conditions, and other personal traits affected vaccine acceptability.

*ask you when you are here, when getting injected, which one is good for me to be injected with. What is suitable for me? And which one isn’t suitable for me. You see … the community wants to get first-hand information from me. They want to know which one is good, and which one is better.* **Kangemi** CHV **01 Script**

### Theme 2: fear of side effects was a barrier

The fear of side effects was cited as a major hindrance to uptake of the COVID-19 vaccines in both Kilifi and Kangemi. CHVs reported community members’ concerns on potential loss of income while nursing vaccine side effects. Reports of side effects experienced by those who got vaccine shots fuelled the reluctance. Additionally, in both Kilifi and Kangemi, community members were reported at the outset to take a cautious approach. They wanted to see how those who took the vaccine shots were impacted health wise before committing to receiving them. Over time the confidence to take the vaccines increased as the communities did not hear of severe adverse events.

#### Sub-theme 2.1: lost time from work/income loss

One of the biggest fears expressed was that vaccination side effects would cause people to lose time from work and consequently, income. Such anxieties were especially apparent among breadwinners.

**CHV:** *This is because they were saying they are being injected but tomorrow we are not going to be able to go to work.* **Kangemi** CHV **02 Script**

*Interviewer: **What were they saying?***

**CHV:** *That the injection has a lot of side effects in the body when injected.*

*Interviewer:* **Like which one?**

**CHV:** *That the hand was becoming numb …* **Kangemi** CHV **02 Script**

*So, when you are injected, you can stay even a week without doing any work.* **Kangemi** CHV **02 Script**

*Some refused because they were the breadwinners*. **Kangemi** CHV **02 Script**

*Another one could say he was vaccinated, and he experienced dizziness.* **Kangemi** CHV **10 Script**

#### Sub-theme 2.2: reported side effects

There were also concerns about reported vaccine side effects. In one instance, a woman’s menstrual cycle was delayed following vaccination.

*Someone would tell you I was injected, for example, I remember there was a woman who was injected, and her menses delayed…* **Kangemi** CHV **10 Script**

#### Sub-theme 2.3: delayed acceptance to ascertain vaccine impact on others

Some respondents reported waiting to see how the vaccines affected others before accepting vaccination themselves. This hesitation can be explained by worries about potential adverse effects. Those initially skeptical about the vaccine became less so as more of the community members received vaccinations and shared their experiences.

*They wanted others to be vaccinated first.* **Kangemi** CHV **10 Script**

…*but when now many people were getting vaccinated other people gained confidence. Now that the bad things that were to happen to them did not occur…* **Kilifi CHA 03**

### Theme 3: misinformation was widespread

Vaccine education and promotion efforts by CHVs were also generally hindered by misinformation. The CHVs gave accounts of how rumors were spreading within their communities regarding the vaccines being linked to causing infertility. Some community members believed that the vaccines caused ill-health and death following rumors or experiences of adverse events. Further, there were some who denied that COVID-19 existed, subscribing to different conspiracy theories.

#### Sub-theme 3.1: vaccine as a cause of infertility

There were several examples of misinformation affecting vaccine uptake. Some community members believed there was a link between vaccination and infertility or that vaccines were being used as a family planning tool. Misinformation caused reluctance and resistance to vaccination.

*There are others who said that when injected you will lose your ability to have children*. **Kangemi** CHV **02 Script**

*… at first, the community denied the vaccination. They thought that it was a family planning method, or it was just something that will kill them.* **Kangemi** CHV **03 Script**

*Yes, there were many challenges with vaccination. [Inaudible] several drives, vaccination drives within the village, within the community. It was because the approach was different, the perception of the COVID jabs was not positive. They took it the African way. Some could say that it’s a way of family planning, such kind of funny [inaudible]. That women will not have children, the men will not function, you understand?* **Kangemi** CHV **06 Script**

*Yeah. Because they were assuming that we are forcing them to get the vaccine, like, they were saying the vaccines are for family planning method and now we were forcing them. They were not believing a vaccine for COVID-19. So, you could get insults, someone could just stop you when talking and then walk away.* **Kangemi** CHV **03 Script**

*Most people they think they are being done the family planning methods against their will. Yes …* **Kilifi** CHV **05 Script**

#### Sub-theme 3.2: interference with well-being and death

Some believed that the vaccine interfered with the normal functioning of bodily processes and would result in chronic illness or death. These perceptions increased vaccine hesitancy. Further, some claimed to constantly feel unwell after being vaccinated, increasing reluctance among other community members.

*Some they give me something like… {That injection is interfering with the body parts}. That some things have changed. They feel they are sick all the time but for me I remember when I was given the first dose I felt like {I had caught a slight cold}…kind of*. **Kangemi** CHV **04 Script**

Others attributed death of friends to the vaccine.

*Yes, we were and even, now even that was an issue because some people were refusing. Yes, you will tell some people go and get the Moderna and they would say ‘me I don’t want that one’ or ‘if my friend had not received the vaccination he wouldn’t have died’. You see they start telling stories about their friends, about other people.* **Kangemi** CHV **05 Script**

Further, there were rumors of death due to boosters that also dissuaded community members from taking these up.

*The booster has been an issue in the community. People were saying if you take a booster you die, like some of the people who were taking the booster, they had other underlying issues like one of our CHVs, we lost one of our CHVs. Now the rumour had it that the booster is the one that made the CHV to die. Now, the uptake of boosters is low because many people do not want to take them. They believe that when they take the booster vaccine, they're going to die.* **Kangemi** *CHV* **03 Script**

#### Sub-theme 3.3: non-existence of COVID-19

Some members of the community believed COVID-19 was a hoax or scam, which negatively affected their adherence to the global guidance on vaccination. Reasons for this denial of COVID-19 included false information and conspiracy theories, and a more pervasive lack of trust in official sources.

*Some declined the facts while some said that the COVID-19 just a scam, they don't believe in it. So, they feared to come to the facility to test this, they hid the signs of COVID-19. Yeah, it was not that easy the during the COVID-19 period. **Kangemi** CHV **03 Script***

### Theme 4: lack of trust in the government hindered uptake

Participants’ narratives shed light on the issues of trust in the government, especially given that the vaccine roll-out was conducted in the lead up to the presidential elections. Some refused vaccine shots because they believed they were a tool used by the government to manipulate them into somehow ‘involuntarily voting’ or being ‘involuntarily sold’. These perspectives on the politicization of the vaccine uptake were pronounced in rural Kilifi.

#### Sub-theme 4.1: rumors of voting through the COVID-19 vaccine

Analysis indicated a high degree of suspicion in these communities that they were being tricked into voting by accepting the vaccine. This was due to the vaccine roll-out occurring close to the election period. This lack of faith in the government’s motives may have been a factor in vaccination reluctance.

*That is the only challenge, and I don’t have any other. That was the challenge I experienced, you tell someone to go and get vaccinated and others would say that is COVID, another one tells you that we are voting through getting the vaccine.* **Kilifi** CHV **02 Script**

#### Sub-theme 4.2: government accused of selling citizens

Some members of the community believed the distribution of COVID-19 vaccines was tied to nefarious purposes, accusing the government of selling its people. This was linked to a broader lack of faith in the government’s motives.

*That you should go and get the vaccine because even I am telling you that I have received the vaccine. When you get that, even if one in your houses gets it, it won't attack you much because you'll have the vaccine that protects you. But people started to say that if the vaccine is given, they are looking for a way to sell people of the country.* **Kilifi** CHV **07 Script**

### Theme 5: CHVs’ efforts were a facilitator to increased uptake

Overall CHVs, mentioned being prioritized for vaccination and used this as an opportunity for positive role-modeling in their communities, encouraging others to follow suit. They also highlighted how crucial their vaccine education and promotion efforts were in increasing uptake in their communities, with most of them reporting vaccine uptake rates of 80% or more among their catchment communities.

#### Sub-theme 5.1: CHVs prioritized for vaccination

As frontline health workers with increased exposure to SARS-CoV-2, CHVs were prioritized for vaccines. CHVs reported that they saw the importance of leading by example, as they could hardly ask others to be vaccinated if they had not accepted. CHVs are dependable members of the community who can communicate with local people and provide information regarding the effectiveness and safety of COVID-19 vaccines. By serving as role models for the community and advocating vaccination, CHVs appeared to have had a significant influence on vaccine uptake.

*they were treated as health workers, so they were given priority for the vaccination so a large percentage of them, approximately ninety six percent have been vaccinated if not a hundred*. **Kangemi CHA 01**

*And because I'm among the staff, so we followed that category. And I mobilize the community on the importance of getting that vaccine.* **Kilifi** CHV **10 Script**

*And you can’t mobilize people to get vaccinated once you are not vaccinated yourself so it’s for them to lead by example.* **Kilifi** CHA **02**

*Actually, in the beginning, people were fearing to come. So, at the facility the number was so low but after getting out* **Kangemi** CHV **01 Script**

#### Sub-theme 5.2: CHVs increased vaccination uptake

CHVs worked hard to increase vaccine uptake. They also tracked uptake rates, with most citing that rates within their communities were 80% and above. CHVs promoted vaccination uptake by conducting community sensitization, delivering information, and dispelling myths and misconceptions.

*The turnout was quite good. Yes, I think it was almost 80%. From this time that there was that fear maybe those that are vaccinated what will happen but through sensitization and creation of awareness isolation and awareness getting them the right information there was a positive reaction from the members of their community, of my households Yes. I think most of them are vaccinated. okay.* **Kangemi** CHV **09 Script**

*In my area I have tried so much, up to 80% have received the vaccination.* **Kangemi** CHV **02 Script**

*At start at my community households only thirty had been vaccinated at first. And we had sensitized them and through the TV … the social media that the importance of vaccination. So, they accepted later on, but at the beginning they declined*. **Kangemi** CHV **03 Script**

*They went for the COVID vaccine…at least three quarters of my households those that I am aware of COVID-19 vaccines and how they can prevent themselves from COVID-19.* **Kangemi** CHV **04 Script**

*Okay, the uptake as of now, many community members have been vaccinated. So up to now, the turnout is good. Just a few now come in because we are still vaccinating up to now.* **Kangemi** CHV **03 Script**

*The turnout was quite good. Yes, I think it almost 80%…* **Kangemi** CHV **09 Script**

*Their areas, okay, the uptake, it’s like eighty percent, let’s work with percentage they have not achieved one hundred because we have the myths and misconceptions in the community …* **Kangemi CHA 03**

However, despite their efforts, CHVs reported that some community members still refused to be vaccinated.

*There were still others who accepted, and others refused…* **Kangemi** CHV **10 Script**

*Wow, That one was also another big challenge for some were appreciative while other refused until today*…**Kangemi** CHV **02 Script**

*They also have to educate on which vaccines are present within the facility to create options for them, these vaccines are administered within this period of time, just quality information about the vaccines.* **Kangemi** *CHA 04 Script*

## Discussion

The aim of this qualitative study was to understand the experiences of Community Health Volunteers (CHVs) in promoting COVID-19 vaccine uptake in two areas in Kenya. Our findings revealed five main themes of these experiences: vaccine preferences, fears of side effects, misinformation, lack of trust in government, and vaccine uptake.

Vaccine preference was a major factor influencing uptake in the communities studied. Understanding attitudes toward vaccines requires having a grasp of vaccine preferences. Anxiety regarding certain vaccine types can negatively affect vaccine uptake. Our study demonstrates the many factors that affect vaccine preferences, including fear, false information, and availability. There was a preference for the Moderna vaccine over AstraZeneca and Johnson and Johnson, which were shunned because of misinformed beliefs that they caused death and insomnia, respectively. Myths and misconceptions about side effects also influenced vaccination preference. Misinformation is known to be a major driver of vaccine hesitancy (26). Individuals may avoid vaccination due to fear and reluctance caused by misinformation. To combat this, communication efforts must prioritize the distribution of correct information. Clear communication about vaccination safety, effectiveness, and the rigorous approval process may help improve confidence and dispel vaccine myths.

It was also evident that certain individuals felt ambiguity about whether vaccines were appropriate for them specifically. Recognizing individual concerns and giving personalized information is key to addressing this issue. Building vaccination confidence and resolving doubts among community members depends on reliable sources. Prioritizing tailored communication and clearly addressing individuals’ concerns is crucial in supporting decisions about vaccine uptake. Healthcare providers could assist communities in making informed vaccination decisions by adapting information to their concerns and working with CHVs to provide information about available vaccines, ensuring that information can be understood by community members. There is also a need to maintain a consistent supply of vaccinations to accommodate individual preferences where possible.

As well as affecting vaccine preference, fear of side effects was cited by CHVs as significantly limiting uptake more generally. This finding is in line with those of other studies which have illustrated how vaccine hesitancy in LMICs was fuelled by concerns about side effects, with 57% of people in Kenya and 30% of South Africans reported to have refused the vaccine for this reason (27, 28). Such concerns have also caused hesitancy in other countries (29–31). In our study, the possibility of infertility was one side effect that caused particular concern. A study in Tanzania found that worries over infertility as a negative consequence of the vaccines contributed to COVID-19 vaccine hesitancy (32). To address such problems, focused risk communication and community engagement strategies should be used to dispel misinformation and promote correct information. Further, reassurance could come from community members sharing testimonials about their vaccination experiences. Respected community leaders who openly discuss their experiences and support vaccination campaigns can foster trust and inspire others to do the same increasing vaccine uptake.

Another fear cited in our study is that of lost income through missed days of work (27). Communities needed assurance that short-term discomfort from side effects was minor in relation to the protection conferred against COVID-19’s impact on health and well-being. To address this, public health officials should work with employers to provide clear information on vaccine safety and offer assistance for people experiencing side effects. It also points to the need to aid people who might have to miss work due to side effects. This could take the form of compensated sick time or modified work schedules.

We found that some individuals postponed being vaccinated to observe reactions in others. Countering this apprehension requires public health initiatives that promote positive vaccination experiences, with individuals who have been vaccinated being encouraged to share their experiences to increase trust and confidence in the vaccine. Community leaders and influencers may play an important role in setting a good example for others to follow. Further, it is crucial to communicate possible adverse reactions and the steps that should be taken in such instances.

Misinformation was another factor preventing vaccine uptake. Reports indicated that many believed COVID-19 to be a hoax, an observation that is consistent with findings from Tanzania, Turkey, and the U.S. (31–33). To address misinformation and disinformation, public health officials and healthcare practitioners must prioritize the distribution of correct information. Incorrect beliefs can be dispelled by working with community leaders and influencers to deliver clear information regarding vaccination benefits and safety. Open forums and educational workshops, for example, can address concerns and give evidence-based responses to enable informed vaccination decision-making. Accurate information, community participation, and compassionate communication can help improve public health outcomes.

Lack of trust in the government was also a major hurdle, according to CHVs. This is consistent with findings from a study conducted across countries which found Kenya to rank highest in the correlation between lack of trust in the government and non-acceptance of the vaccine (34). A similar association has been shown in China (35, 36). Belief in conspiracy theories, such as vaccines being state-backed interventions, decreased trust in vaccines and restricted uptake (37, 38). It is important that trust between government and citizens is continuously cultivated and that steps are taken to address sources of mistrust. Campaigns to depoliticize vaccination can help restore faith in government action. Relationships between communities and trusted workers such as CHVs can be vital in this endeavor.

Findings indicated that CHVs were rightly prioritized for COVID-19 vaccination alongside other frontline health workers. This is consistent with WHO guidance (4). CHVs were actively involved in promoting vaccine uptake, with most reporting uptake rates of 80% and above in their communities. Their participation in promoting vaccination, conducting sensitization programs, and providing accurate information had a substantial influence. Through being prioritized, they were able to role-model being vaccinated and encourage communities to also do the same. Similarly, in other contexts, by highlighting the importance of COVID-19 vaccines and showing confidence in them, CHWs positively impacted community uptake (4, 14), with evidence supporting that vaccinated healthcare workers are more likely than unvaccinated ones to encourage the public to receive vaccination (14, 39). CHVs also engaged with the community as trusted members to deliver accurate information on COVID-19 vaccination uptake and sharing success stories of CHVs who had received the vaccine was important in increasing vaccination uptake and counteracting unfavorable narratives. An implication is that vaccination hesitancy can be tackled through focused communication techniques.

## Strengths and limitations

This study contributes to a significant research gap regarding LMICs such as Kenya where the experience of CHVs in promoting COVID-19 vaccine uptake is not well-studied. The questions covered during the interviews enabled establishment of rapport, and guided robust understanding of the overall work of CHVs during the pandemic and how they experienced their work with communities specifically in promoting COVID-19 vaccine uptake. Our findings have the potential to guide the development of risk communication and community engagement materials and inform guidelines and policies on vaccine uptake and acceptance, not only during pandemics but also in routine immunization programs. Recruitment of urban and rural participants is also a strength of our study and for each theme, a comparison of the findings between the two distinct geographical areas has been made.

Despite these strengths, interpretation of our findings may be limited by the study being conducted in just two of 47 counties in Kenya. Also, there is diversity in the experiences of CHVs and our findings may not reflect the perspectives of all CHVs across the country. That said, although findings may not be generalizable to the entire country, they might be transferable. Furthermore, although we did data source triangulation between CHVs and CHAs, a limitation was the lack of triangulation to corroborate findings from CHVs with those from community members. Representation of communities’ own narratives would have deepened understanding of how vaccine education and promotion was experienced by community members and would have enhanced the credibility and validity of our study findings. In addition, a limitation to note is that there was no pre/post-intervention study or measurement here to evaluate whether use of CHVs was associated with improved vaccine coverage.

Future research should focus on building upon the findings of this exploration, potentially deploying a mixed methods approach. Further, future studies should focus on co-creating and testing the feasibility of risk communication and community engagement models led by CHVs and their communities, that have a higher likelihood of success in increasing adoption of global guidance and addressing the barriers uniquely identified in the pandemic context.

## Conclusion and recommendations

This study affirms the important role CHVs played in mobilizing communities to take up primary vaccines and boosters during the pandemic through vaccine education and promotion. CHVs acting as role models by receiving vaccinations first was a particularly strong driver of community uptake. Our findings support the need to tailor risk communication and community engagement to address myths and misconceptions, while also leveraging factors that can promote vaccination uptake such as the established positive and trusted relationships with CHVs. Further, the findings demonstrate the importance of prioritizing CHVs in the planning and implementation of future vaccination initiatives in Kenya and similar countries. Finally, the findings reveal underlying issues of public trust toward vaccination drives. This points to the crucial role of continual education and awareness raising in communities on the importance of vaccines, increasing the likelihood of vaccine uptake in situations of health crisis such as COVID-19.

## Data availability statement

The original contributions presented in the study are included in the article/supplementary material, further inquiries can be directed to the corresponding author.

## Ethics statement

The studies involving humans were approved by Aga Khan Kenya’s Institutional Scientific and Ethics Review Committee [Ref: 2020/IERC-89(v3)] and the National Commission for Science Technology and Innovation (EOP/NMS/HS/088). The studies were conducted in accordance with the local legislation and institutional requirements. The participants provided their written informed consent to participate in this study.

## Author contributions

CS: Conceptualization, Formal analysis, Funding acquisition, Methodology, Validation, Writing – original draft, Writing – review & editing. PK: Data curation, Formal analysis, Investigation, Validation, Writing – review & editing. IK: Methodology, Validation, Writing – review & editing. SS: Conceptualization, Funding acquisition, Methodology, Supervision, Validation, Writing – review & editing.
